# Correlation of regional deposition dosage for inhaled nanoparticles in human and rat olfactory

**DOI:** 10.1186/s12989-019-0290-8

**Published:** 2019-01-25

**Authors:** Lin Tian, Yidan Shang, Rui Chen, Ru Bai, Chunying Chen, Kiao Inthavong, Jiyuan Tu

**Affiliations:** 1 School of Engineering – Mechanical and Automotive, RMIT University, Bundoora, VIC Australia; 2 CAS Key Lab for Biomedical Effects of Nanomaterials and Nanosafety & CAS Center for Excellence in Nanoscience, Beijing Key Laboratory of Ambient Particles Health Effects and Prevention Techniques, National Center for Nanoscience and Technology of China, Beijing, China; 3 Key Laboratory of Ministry of Education for Advanced Reactor Engineering and Safety, Institute of Nuclear and New Energy Technology, Tsinghua University, Beijing, China

**Keywords:** Inhalation toxicity, Neurotoxicity, Nanoparticles, Nasal olfactory, Olfactory pathway, Olfactory deposition, Human and rat interspecies extrapolation

## Abstract

**Background:**

Nose-to-brain transport of airborne ultrafine particles (UFPs) via the olfactory pathway has been verified as a possible route for particle translocation into the brain. The exact relationship between increased airborne toxicant exposure and neurological deterioration in the human central nervous system, is still unclear. However, the nasal olfactory is undoubtedly a critical junction where the time course and toxicant dose dependency might be inferred.

**Method:**

Computational fluid-particle dynamics modeling of inhaled nanoparticles (1 to 100 nm) under low to moderate breathing conditions (5 to 14 L/min – human; and 0.14 to 0.40 L/min – rat) were performed in physiologically realistic human and rat nasal airways. The simulation emphasized olfactory deposition, and variations in airflow and particle flux caused by the inter-species airway geometry differences. Empirical equations were developed to predict regional deposition rates of inhaled nanoparticles on human and rat olfactory mucosa in sedentary breathing. Considering, breathing and geometric differences, quantified correlations between human and the rat olfactory deposition dose against a variety of metrics were proposed.

**Results:**

Regional deposition of nanoparticles in human and the rat olfactory was extremely low, with the highest deposition (< 3.5 and 8.1%) occurring for high diffusivity particles of 1.5 nm and 5 nm, respectively. Due to significant filtering of extremely small particles (< 2 nm) by abrupt sharp turns at front of the rat nose, only small fractions of the inhaled nanoparticles (in this range) reached rat olfactory than that in human (1.25 to 45%); however, for larger sizes (> 3 nm), significantly higher percentage of the inhaled nanoparticles reached rat nasal olfactory than that in human (2 to 32 folds). Taking into account the physical and geometric features between human and rat, the total deposition rate (#/min) and deposition rate per unit surface area (#/min/mm^2^) were comparable for particles> 3 nm. However, when body mass was considered, the normalized deposition rate (#/min/kg) in the rat olfactory region exceeded that in the human. Nanoparticles < 1.5 nm were filtered out by rat anterior nasal cavity, and therefore deposition in human olfactory region exceeded that in the rat model.

**Conclusion:**

Regional deposition dose of inhaled nanoparticles in a human and rat olfactory region was governed by particle size and the breathing rate. Interspecies correlation was determined by combining the effect of deposition dosage, physical\geometric features, and genetic differences. Developed empirical equations provided a tool to quantify inhaled nanoparticle dose in human and rat nasal olfactory regions, which lay the ground work for comprehensive interspecies correlation between the two species. Furthermore, this study contributes to the fields in toxicology, i.e., neurotoxicity evaluation and risk assessment of UFPs, in long-term and low-dose inhalation exposure scenarios.

## Background

Neurological disorders, such as Parkinson’s and Alzheimer’s disease, are suspected to be related to long-term brain accumulation of toxicants, which may lead to a gradual progression and staged neurological responses [1]. In such a hypothesis, chronic low-dose exposure and prolonged brain accumulation of exogenous airborne ultrafine particles (UFPs) are critical to define the neurodegeneration. The brain is protected by the blood-brain barrier (BBB), which is a tight junction that prevents toxicants from entering [2, 3]. However, particle transport via nose-to-brain route has attracted increased attention based on the findings that exogenous materials can bypass the BBB via the olfactory pathway. Additionally, researchers have established a link between ambient air pollution, olfactory function and the potential contribution to cognitive function [3–8]. The findings suggested negative impact to human olfactory function and plausible toxicological mechanisms contributing to human cognitive impairment by environmental exposure to ambient pollutants via the olfactory pathway.

Translocation of solid ultrafine particles, in the form of 30 nm polio virus through the olfactory pathway, was observed more than half century ago (1940s) in chimpanzees and rhesus monkeys with intranasal instillation [9, 10]. A remarkable particle transmission velocity of 2.4 mm/h in olfactory axons was proposed. The first conclusive findings of brain translocation of inhaled gold nanoparticles were captured by De Lorenzo et al. (1970) in squirrel monkeys. Captured sequential images depicted the entire process including nanoparticle deposition onto olfactory mucosa, particle uptake via the olfactory rod, retrograde translocation within olfactory dendrites, anterograde movement in the axoplasma (along olfactory nerve), entrance into the olfactory bulb, and arrival in mitral cell dendrites within the brain. A neuronal transport velocity of 2.5 mm/h was calculated [11]. Clear evidence was also presented by Gianutsos et al. (1997) where unilateral intranasal instillation of manganese chloride in the right nostril of the rats resulted in elevated manganese levels in the right olfactory bulb and tubercle, while the left brain was unaffected [12]. Uptake of manganese, cadmium, nickel, mercury, and cobalt nanomaterial via rat olfactory pathways were also demonstrated in a series of recent studies [13–16]. These observations support the fact that nasal olfactory deposition plays a key role in brain neurotoxicant accumulation via the nose-to-brain pathway.

While the reported evidences were predominantly in animal subjects, olfactory pathway is considered a functional route for brain uptake of inhaled nanoparticles in humans. Using single photon emission computed tomography (SPECT), X-ray computed tomography (CT), and MRI (magnetic resonance imaging), Shiga et al. (2010) observed appreciable movement of thallium to the olfactory bulb in human volunteers following nasal administration. The observation agreed with the transport kinetic properties and olfactory delivery route measured in rodent studies [17, 18]. Comparative studies between seasoned welders and normal controls also suggested olfactory pathway as a viable route for brain accumulation of manganese through inhaled welding fumes [19].

While there remain significant knowledge gaps before researchers fully understand the relationship between toxic substances (e.g., heavy metals) and neuron functional degeneration in human central nervous system, the nasal olfactory is undoubtedly a critical junction where the time course and dose dependency might be inferred. Based on rat whole body inhalation study, Oberdorster et al. (2004) reported an estimated 20% migration of deposited nanoparticles (36 nm carbon) from olfactory mucosa in the nasal chamber to the olfactory bulb of the central nervous system [20]. This suggests knowledge of dose on olfactory mucosa can lead to statistical analysis linking brain uptake efficiency via the olfactory pathway.

Due to technical difficulties, quantitative measures of nanoparticle deposition onto nasal olfactory mucosa are not experimentally available in either animal or human subjects. Quantitative assessments were scarcely provided by very few computational studies in literature. Garcia and Kimbell (2009) were the first to propose a rat olfactory deposition equation based on a computational investigation of inhaled nanoparticles (1 to 100 nm) at 1x, 1.5x and 2x the resting breathing rates [21]. A similar approach was applied in the human nasal olfactory models for breathing rates of 15 to 30 L/min by the same group [22]. There is no other reported study quantitatively assessing nasal olfactory deposition in either human or animal subject. However, there is a wide range of airflow studies, particle transport and depositions in rat and human in the entire nasal cavity. Kelly et al. (2004), Cheng et al. (1995, 1990), Swift et al. (1992), Gerde et al. (1991) and Wong et al. (2008) experimentally measured the total deposition of ultrafine particles in rat and human nasal airway replicas [23–28]. Computationally, Subramaniam et al. (1998), Matida et al. (2003), Xi and Longest (2008), Inthavong et al. (2014), Ge et al. (2012), and Zamankhan et al. (2006) applied CFD (Computational Fluid Dynamics) approaches in the human nasal/head airways for airflow and particle transport analysis [29–34]. More recently, there was a focus on deposition flux and intensity in the entire nasal cavity, where Shang et al. (2015) and Dong et al. (2016, 2018) numerically performed comparative studies of olfactory deposition between human and the rats [35–37]. Tian et al. (2016) calculated the deposition of inhaled welding fume agglomerates onto human olfactory mucosa at a breathing rate of 30 L/min [38]. In summary, prior studies mainly focused on the entire nasal cavity, where transport processes in the nasal olfactory was either ignored or only briefly discussed.

To fill the gap, Tian et al. (2017) performed a comprehensive numerical analysis on airflow pattern and nanoparticle flux in a human nasal cavity and olfactory region, where key factors contributing to olfactory deposition at low to moderate breathing rates were investigated [39]. The study found olfactory deposition efficiency was extremely low (< 3.5%) and showed distinctive variation in high diffusivity regions compared to the entire nasal cavity. The results showed an exponential decay correlation as a function of the breathing rate and particle diffusion coefficient, frequently seen in the entire nasal cavity, was unsuitable to characterize olfactory nanoparticle deposition. Furthermore, the initial locations of olfactory deposited nanoparticles predominantly originated from the superior nostril inlet along the nasal septum side.

Based on the work of Tian et al. (2017) [39], this study extends past computational investigations to rat nasal olfactory, with the aim to provide an understanding of the physical process for nanoparticle transport and deposition in the Sprague-Dawley rat. Of particular significance is a focus on olfactory particle deposition, and interspecies correlation between human and laboratory animal subjects, which has been extremely lacking. This study performed simulations of inhaled nanoparticles (1 to 100 nm) under low to moderate breathing conditions (0.14 to 0.40 L/min) in a physiologically realistic rat nasal airway replica. The result was compared to that in human (Tian et al. 2017, [39]) and interspecies correlation was examined. Furthermore, the current study proposed two empirical equations that predict local deposition rate of inhaled nanoparticles in human and rat olfactory mucosa under sedentary breathing. The empirical equations enable quantification of long-term low-dose exposure to nano toxicants through the olfactory pathway. This also allows correlations between human and rat olfactory deposition which is critical for accurate interspecies data extrapolations. The current findings are valuable to the toxicology community in context of the growing interest in neurotoxicity of inhalation exposure via the olfactory pathway.

## Methods

### Nasal and olfactory airway modeling for human and rat

**Fig. 1:**
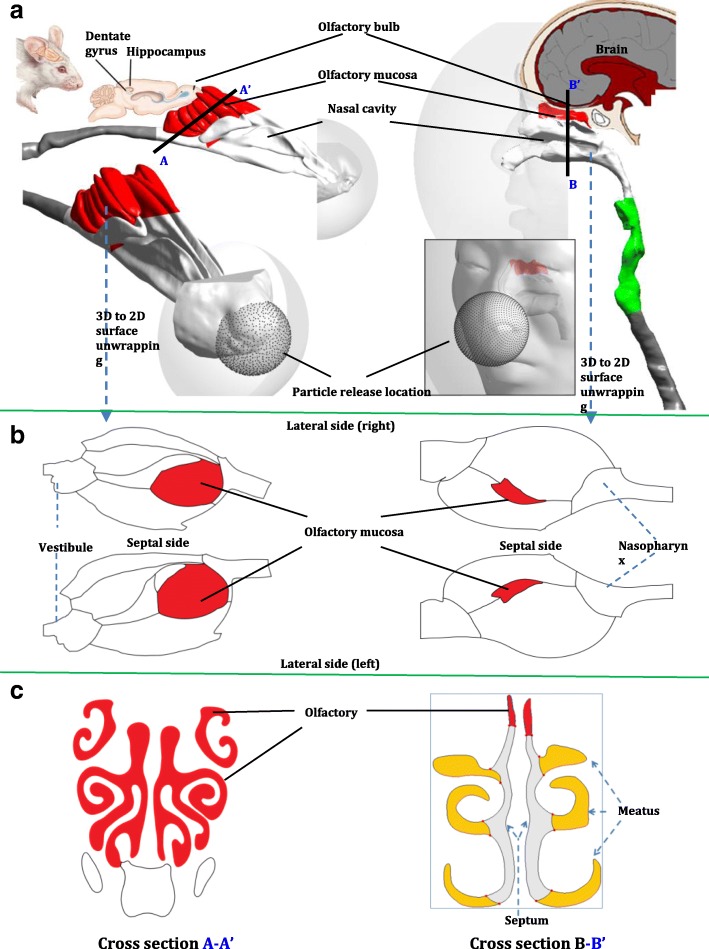
Human and rat nasal and olfactory model: (**a**) upper respiratory airways, olfactory brain pathways, and particle release profile; (**b**) 2D unwrapped nasal cavity surface model. (Brain images: Univ ersity of Calgary, 2006 (human), The Scientist, 2015 (rat)); (**c**) sample cross section of nasal and olfactory channel

Computational models of a human (48-year old healthy male, Asian, 88 kg in body weight) and rat (12-week old male Sprague-Dawley rat, 400 g body weight) upper respiratory airway including of facial features, nasal cavity, larynx, and trachea were developed from CT scans (Fig. 1a) [35]. Each model was connected to form a contiguous path from the external space, through the nostrils and main nasal passage, and exiting at the larynx. The larynx region was extended to the trachea to allow sufficient flow recovery and improve numerical convergence in the CFD solution. A realistic human or rat face was connected which was exposed to the external surroundings containing airborne particles from the ambient environment. Further details of the model reconstruction and verification can be found in the prior work [35, 36, 39–42].

To visualize the complex nasal surfaces, 2D unwrapped nasal cavity models, showing details of the relative position and surface feature for the human and rat olfactory mucosa was created (Fig. 1b). The initial 3D model was first sliced along the centerline of the nasal passage floor, at the lateral and septal wall interface. Then the 3D surface coordinates were transformed into a new set of 2D coordinates, representing an unwrapped surface. Layout of the top and bottom boundaries represents the initial centerline (sliced along the nasal floor), while left and the right boundaries represent the nostril and nasopharynx respectively. Details of surface-mapping technique to transform nasal cavity morphology from 3D onto a planar 2D domain can be found in the initial work of Inthavong et al., 2014 [29]. The olfactory region was highlighted in red to emphasize its relative position in the nasal cavity (Fig. 1).

Fluent Meshing (ANSYS 18.0) was used to generate the computational mesh. A new polyhedron scheme was employed with minimum cell dimension of 1 mm filled the main airway passage and maximum cell dimension of 10 mm filled the external domain. A highly dense 5 prism layers of hexahedron elements in normal direction of the wall was attached to resolve the near wall features. The resulting mesh consisted of 1.2 million elements for the human, and 2.1 million cells for the rat. Mesh independency was conducted and achieved at the specified size. Further details of the computational model could be found in [39].

### Fluid flow simulation

The airflow was simulated using ANSYS-FLUENT18.0 assuming steady flow. The ambient environment was set to atmospheric pressure and inhalation was initiated by a negative pressure difference between external environment and the airway exit. This allowed the ambient flow field to be influenced only by the inhaled air. The continuity and momentum equation of the fluid flow are:

1 $$ \frac{\partial }{\partial {x}_i}\left(\rho\;{u}_i\right)=0 $$

2 $$ \rho\;{u}_j\frac{\partial {u}_i}{\partial {x}_j}=-\frac{\partial p}{\partial {x}_i}+\frac{\partial }{\partial {x}_j}\left[\mu \frac{\partial {u}_i}{\partial {x}_j}\right] $$

where *ρ*, *u* and *p* are density, velocity and pressure of the air, respectively. A second order upwind scheme was used to approximate the momentum equation, while the pressure-velocity coupling was handled through the SIMPLE method. Further details of the fluid flow modeling are given in [43].

### Particle simulation

The Lagrangian particle tracking method was used where individual particle trajectories were computed. The particle equation is:

3 $$ \frac{du_p}{dt}=\frac{1}{C_c}{F}_D+\frac{g\left({\rho}_p-\rho \right)}{\rho_p}+{F}_L+{F}_B $$

where ***u***_***p***_ is the particle velocity, *t* is the time, ***g*** is the gravitational constant, *ρ*_*p*_ is the particle density. In this study, both gravitational and buoyancy forces can be neglected. ***F***_***D***_ is the drag force given by *18 μ(****u***_***p***_*-****u****)/(d*^*2*^*ρ*_*p*_*),* with *d* being the particle diameter, and *C*_*c*_ the Cunningham correction given by:

4 $$ {C}_c=1+\frac{2\lambda }{d}\left(1.257+0.4{e}^{\left(-1.1d/2\lambda \right)}\right) $$

here λ is the molecular mean free path. ***F***_***L***_ in Eq. (3) is the Saffman lift force, and ***F***_***B***_ is the Brownian diffusion force with amplitude of $\zeta \sqrt{\pi {S}_0/\Delta t}/\varsigma$ is a zero mean, unit variance independent Gaussian random numbers. ∆t is the time-step for particle integration and *S*_*o*_ is a spectral intensity function [44]:

5 $$ {S}_o=\frac{216 vkT}{\pi^2\rho\;{d}^5{\left(\frac{\rho_p}{\rho}\right)}^2{C}_c} $$

*ν* is the fluid kinematic viscosity, *k* is the Boltzmann constant, and *T* is the absolute temperature of the inspiratory air in the nasal cavity. The simulation was carried out with ANSYS-FLUENT18.0 discrete phase model (DPM).

Particles were uniformly released from a hemispherical surface (of radius 3 cm for human, and 0.5 cm for rat) with the center at the nose tip (Fig. 1a), resembling the release condition of Doorly et al. (2008) [45]. Statistically independent 100,000 mono-dispersed particles of 1, 1.1, 1.2, 1.3, 1.4, 1.5, 1.7, 2, 3, 5, 10, 15, 20, 30, 40, 50, 70 and 100 nm, were released. Deposition onto the respiratory walls occurred when the particle was within one radius distance away from the surface.

### Particle flux

Particle spatial distribution across the nasal airway passage was described using a particle flux (*f*) defined as the number of particles passing through a cross-sectional area within the nasal cavity. *f* is normalized with the total number of particles entering the nasal cavity, therefore is equal to 1.0 at the nostril inlet. The particle flux along the transport route flux is expected to decrease as particles deposited along the transport route. This can shed light on particle bulk movement and likelihood of olfactory deposition. Accordingly,

6 $$ f\left(\overrightarrow{r}\right)=\frac{\# particles\; per\; unit\kern0.17em cross\kern0.17em sectional\kern0.17em area}{\# particles\kern0.17em entering\kern0.17em nasal\kern0.17em cavity} $$

7 $$ \underset{Nostril}{\int }f\left(\overrightarrow{r}\right) dA=1.0\;\mathrm{and}\int f\left(\overrightarrow{r}\right) dA\le 1.0\kern0.24em \mathrm{on}\ \mathrm{subsequent}\ \mathrm{cross}\ \mathrm{sections}. $$

$\overrightarrow{r}$ is the position vector on cross sections with area element being *dA* in above equations.

## Results

### Human and rat nasal and olfactory airway model comparison

The human nasal olfactory is located in the superior main nasal passage, while for the rat it is predominantly located in the posterior half of the nasal cavity (Fig. 1a). Complex overlapping folds and multiple airway branching patterns were observed for rat nasal olfactory, while for the human it occupies only a single channel per nostril. This results in a significantly higher proportion of the rat nasal airway containing the olfactory region (Fig. 1c), and a substantially larger olfactory mucosa surface area compared to the human model (Fig. 1b).

The physical and geometric features of human and rat nasal models were compared in Table 1. The human olfactory region had a surface area of 2097 mm^2^ and occupied 10.5% of the total nasal cavity [35]. The olfactory channel (Fig. 1c) was about 1–2 mm wide. Comparatively, the rat nasal olfactory had a surface area of 1294 mm^2^, however, occupied 55.6% of the total nasal cavity and its olfactory channel was approximately 0.5 mm wide.

**Table 1:** Physical and geometric features of human and rat models

	*Human*	*Rat*	*Rat/Human Ratio*
Body Mass (kg)	88	0.4	0.0045
Nasal Surface Area (mm^2^)	19,882	2325	0.117
Olfactory Surface Area (mm^2^)	2097	1294	0.617
% of Olfactory to Nasal Surface Area	10.5	55.6	5.295

Table 2 summarizes reported data of nasal surface features for rat and human subjects. Male CrlBr (Fischer-344) rats of body weight 288 g were used in the study of Gross et al. (1982), while a male F344 rat of 315 g was employed by Schroeter et al. (2008), and Garcia and Kimbell (2009) [21, 46, 47]. Gross et al. (1982) provided detailed morphometric analysis of rat nasal cavities, at two age groups and indicated that the nasal cavity surface lined by the olfactory epithelium were similar at both ages. Due to uncertainty of the precise location of the human olfactory epithelium, and also to facilitate comparison of dose estimates, Garcia et al. (2015) provided 3 human olfactory scenarios (detailed in Table 2 [22]). The original model of Garcia et al. (2015) was built from CT scans of a 37-year-old woman, and 2 variants of this model was obtained by halving and doubling the original mapped olfactory region. The models in this study were comparable with the reported percentage values of olfactory to total nasal surface area, where the variation was in accordance to inter-subject variations. Although morphological differences between individual models lead to differences in the regional dose exposure, these differences were not accounted for in this study since this study focused on only one comparison using a single nasal cavity for both species.

**Table 2:** Human and rat nasal surface data in literature report

	Human		Rat		
	A [﻿46]	B [22]	A [46]	C [47]	D [21]
Nasal Surface Area (mm^2^)	24,740	20,160	1820	1344	913
Olfactory Surface Area (mm^2^)	2340	560 1120 2200	760	675	371
% of Olfactory to Nasal Surface Area	9.5	2.8 5.6 10.9	41.8	50.2	40.6

^A^Schroeter et al. (2008), ^B^Garcia et al. (2015), ^C^Gross et al. (1982), ^D^Garcia and Kimbell (2009)

### Breathing airflow pattern

The flow was modelled as steady inhalation with the assumption that particle deposition mainly occurred during the inhalation phase [44] (excludes exhalation). The breathing pattern was shown to affect deposition of micron particles between 1 and 5 μm [48]. However, the effect toward nanoparticle deposition is not fully understood. Steady inhalation is predominantly employed for nanoparticle deposition studies in respiratory airways [21, 22, 46]. Sedentary breathing rates (4 to 14 L/min for human, and 0.14 to 0.40 L/min for rat) were assumed where laminar flow conditions were assumed in the nasal cavity.

**Fig. 2:**
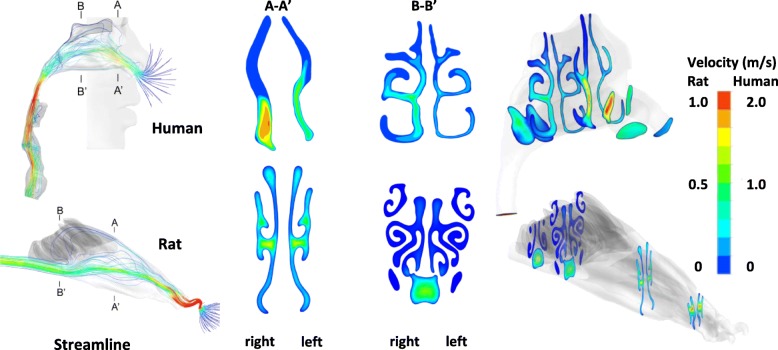
Stream-wise and axial air flow pattern in the nasal and olfactory region at selected locations in human (top row) and rat (bottom row) airways

Figure 2 displays the stream-wise and axial airflow pattern in the nasal and olfactory region at the locations of A-A’ and B-B′ in both airways. Breathing rates of 5 to 14 L/min for human and 0.14 to 0.40 L/min for rat were considered. The corresponding Reynolds numbers (taking hydraulic diameter at the nostril inlets) are given in Table 3. The range of steady flow rates represent resting breathing conditions for human and rat species according to the studies of Kimbell et al. (1997), Kelly et al. (2004), and Schroeter et al. (2008) [23, 46, 49]. Key features of the airflow were influenced by the geometric details of each individual airway, with distinctive variations observed across the two species.

**Table 3:** Airflow rate and Reynolds number in human and rat nasal cavities

*Airflow rate (L/min)*		*Reynolds number*	
Human	Rat	Human	Rat
5	0.14	305	110
7	0.20	427	157
10	0.28	610	220
14	0.40	854	314

Ambient air entered the human nostril in an upward direction and turned 90^o^ entering the middle and inferior nasal meatus before a second 90^o^ turn at posterior nasopharynx. High velocity was observed at the nostril entrance, downstream of the nasal valve and peaked at the larynx. For the rat model, there are sharp turns in the anterior nasal cavity, which produced very high velocity (up to 10 m/s) immediately following the rat nostrils. The remaining rat nasal airway produced smooth streamlines with low to moderate velocities.

In the human model, most of the air passed through the middle and inferior meatus, while very low velocities were found in the superior meatus, where the olfactory region was located. In contrast, most of the air in the rat nasal cavity passed through the middle passage in the anterior half, and then shifted toward the lower respiratory channel before entering deeper airways. This resulted in a very low velocity distribution in rat nasal olfactory, located superiorly in the posterior half of its nasal cavity. The rat nasal olfactory mucosa had prolonged exposure to the airflow compared to the human model, due to significantly larger surface area and sophisticated olfactory structure that occupied a significant portion of the cross sectional area.

The inhaled airborne particles were transported by the moving fluid and regions with higher velocity produced higher particle concentrations. This implied that major air conducting sections (middle and inferior meatus in human nasal cavity, middle and lower passages of the rat nasal cavity) had significantly higher particle concentrations than the nasal olfactory. Significant changes in flow paths induced by the nasal cavity geometry such as sharp turns, sudden contractions or expansion of the cross sectional area, could have intrinsic effects toward particle transport.

Airflow in the olfactory region was very low and free from disruptive changes however, the rat olfactory region was considerably more complex with swirling and overlapping folds, which is unlike the pair of simple channels in the human olfactory region. The human model also exhibits asymmetry between the left and right nasal chambers (right chamber volume > left chamber volume), which is common. In contrast, there is a high level of symmetry in the rat nasal passages.

### Particle flux and spatial distribution

**Fig. 3:**
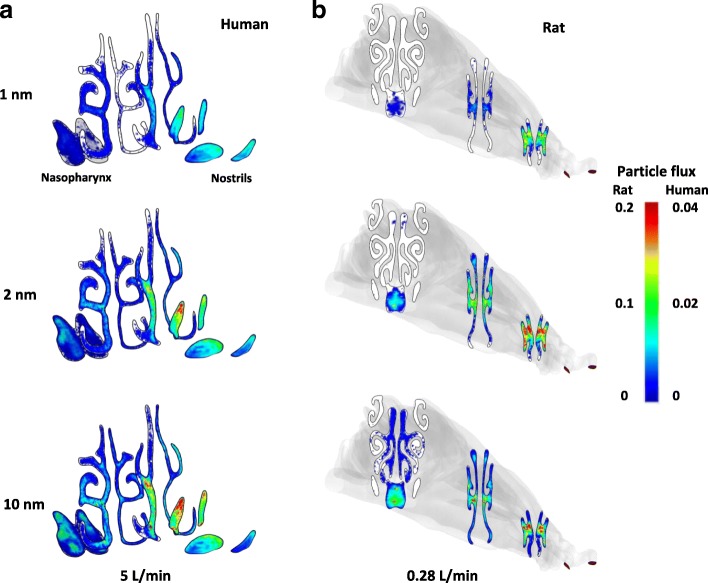
Particle flux at cross sections of the nostrils, anterior and posterior nasal passages in human and rat nasal cavity at breathing rate of 5 L/min (human) and 0.28 L/min (rat). **a** human nasal cavity; **b** rat nasal cavity

Figure 3 shows the particle flux at cross sections of the nostrils, anterior and posterior nasal passages, for 1, 2 and 10 nm at a flowrate of 5 L/min in the human model, and 0.28 L/min in the rat model. For the human model, a clear preferential particle distribution was observed at the nostrils and nasal valves where significantly higher particle concentration was seen near the nose tip and superior section of the openings. This was due to flow acceleration and momentum in both airflow and particles in vicinity of the nostrils, while the concave-shaped nasal cavity floor helped to shield the fluid-particle flow from impinging. A preferential particle distribution was not seen in rat anterior nasal cavity, due to the elongated cone-shaped nose. Additionally, there were sharp turns following the nostrils (Fig. 1).

For both models, particle concentration conformed to the airflow pattern in the main pathways, indicating particle movement was mostly affected by the local environment (Figs. 2 and 3). Significantly higher particle flux was found in the human middle/inferior meatus, while in the rat model, it was found in the middle passages of the anterior half and inferior passages of the posterior half (Fig. 3). The particle spatial distribution was higher on the septal walls, and less on the lateral sides in both species (Fig. 3), while the asymmetric nasal channels in the human model produced higher particle concentration in the right chamber (Figs. 2 and 3). In both models, minimal particle flux was found in the nasal olfactory and the lateral ends of the airway channels. The particle flux decreased in the direction of flow nasal passage due to continuous deposition occurring along the transport route.

Figure 3 shows particle size affected particle flux and spatial distribution (Fig. 3) where larger nanoparticles exhibited stronger concentrations at the nose entrance while smaller sizes were more uniformly distributed (more evident in the human model). This was consistent in the mid and posterior nasal channels, conforming to the airflow patterns (Fig. 2) for both species. Lower particle flux was found for the smaller sized particles, and was most evident in the nasal olfactory where 1 nm particle flux was significantly lower than that of 10 nm particles. Due to the significant cross-sectional area occupied by rat nasal olfactory, the extremely low particle concentration was especially unique. These phenomena were largely related to molecular diffusivity of ultrafine particles – a fundamental mobility property inversely related to the particle size. That is, smaller diameters in the nanometer scale, produces higher diffusivity and dispersion to travel a further distance from the flow path and increase deposition on the walls. Additionally, the anterior particle losses to airway surfaces contribute to the low particle flux observed in the olfactory as seen for the 1 and 2 nm particles in both species (Fig. 3).

The particle transport mechanism and its interaction with the airway geometry could shed light on olfactory deposition. Figure 3 shows the olfactory cavity was hard to reach by inhaled nanoparticles. This is especially prominent for smaller sized particles such as 1 and 2 nm (Fig. 3a and b). The particle flux and spatial distribution show extremely low deposition probability for all sizes onto the olfactory mucosa, where deposition occurs either by: (i) low quantity with higher diffusivity (1 and 2 nm); or (ii) higher quantity but low diffusivity (10 nm). Since the rat olfactory region occupied a significant portion of the nasal cross-sectional area, the relative particle flux through the rat nasal olfactory could exceed that of the human model.

### Particle deposition pattern

**Fig. 4:**
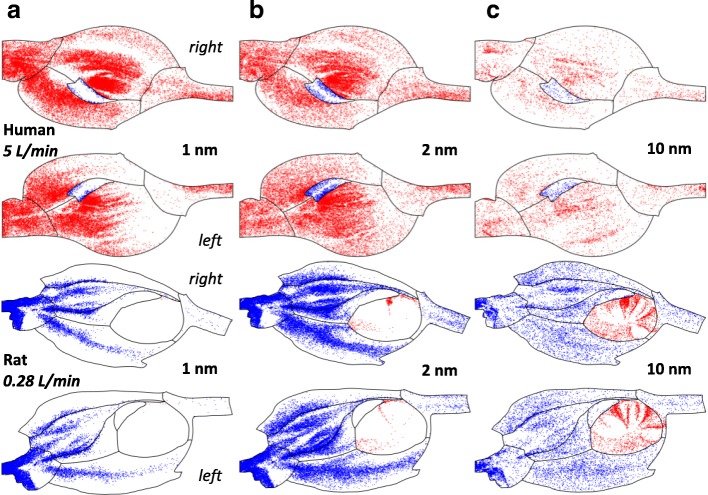
Particle deposition pattern in nasal cavity (human - red, rat - blue) and olfactory (human - blue, rat - red) at 5 L/min. **a** 1 nm; **b** 2 nm; **c** 10 nm

Figure 4 shows deposition patterns for the nanoparticles unwrapped 2D surface of the human and rat nasal cavities at breathing rates of 5 L/min and 0.28 L/min respectively. Particles depositing on the olfactory mucosa were marked in blue while the rest in red for the human, while the opposite color representation was used for the rat. The deposition pattern was influenced by particle size, and there were variations between the models. In the human nasal cavity, high diffusivity particles (such as 1 and 2 nm in Fig. 4) were more likely to deposit with preferential deposition sites at the nasal vestibule, anterior septum, and posteriorly in the converging section of nasopharynx. However, this was dissimilar to the rat nasal cavity, where high diffusivity particles (1 and 2 nm, Fig. 4) were more likely to deposit in the vestibule, septal wall side of the main nasal passage, but was considerably lower in the pharynx. In the human main nasal passage, the majority of deposition occurred in middle meatus, with a small fraction scattered across superior meatus (olfactory region) and the channel floor. In both species, high level of inhomogeneity was observed for 1 and 2 nm particles. For 10 nm particles, the deposition tended to be more homogeneously distributed. The deposition patterns in left and right nasal cavities were asymmetric in the human nasal cavity, with the right chamber captured more (in middle meatus) and the left chamber was slightly more spread. This asymmetric deposition pattern was not found in the rat nasal cavity.

The nasal olfactory mucosa was clearly a difficult region for the inhaled nanoparticles to deposit. The deposition patterns in Fig. 4 show maximum deposition occurred for 2 nm particles in the human model, while 1 and 10 nm deposition was less. The deposition of location of 1 nm and 2 nm in the human nasal olfactory region were similar, concentrated around the border of the olfactory mucosa, while 10 nm particle deposition was uniformly spread over the olfactory region.

In the rat model, 10 nm particles produced maximum deposition while 1 and 2 nm particle deposition was less. There was extremely low deposition of 1 nm particles, but this increased for 2 nm, and increased further for 10 nm particles. There was preferential deposition site was on the septal sides of the rat nasal olfactory. The observed phenomena in both species implied a strong size dependency on olfactory deposition.

Deposition within the inner most region of the human olfactory region is difficult to achieve by the smallest inhaled nanoparticles, which might imply a significant sensitivity to the size of the olfactory mucosa for the extremely small particles (1–2 nm). In the case of 1 nm particles (shown in Fig. 4, human), a slight decrease or increase in the olfactory mucosa area would lead to significant variation in olfactory particle deposition. The sensitivity decreased as particle size increased and is expected to be negligible for ≥10 nm diameter. In this study, the human olfactory mucosa occupied 2097 mm^2^ of the surface area, or about 10.5% of the total nasal cavity. This sensitivity was not seen in rat nasal cavity, where a clear boundary between the rat olfactory region and the main channel was exhibited with distinctive deposition pattern (Fig. 4, rat).

### Particle deposition efficiency and deposition equations

Particle deposition efficiency (DE) is defined as the ratio of the deposited particles in a region to the total number entering the nasal cavity. That is:

8 $$ DE=\frac{\# deposited\kern0.17em particles}{\# particles\kern0.17em entering\kern0.17em the\kern0.17em nasal\kern0.17em cavity} $$

It is an important parameter characterizing the regional filtering capacity and particle penetration rate. In this study, a total of 100,000 mono-dispersed particles were released from a hemisphere (of radius 3 cm for human, and 0.5 cm for rat) with the center at the nose tip (Fig. 1a). Particle deposition efficiency (DE) was calculated as the number of deposited particles divided by the total number of deposited particles and the particles escaped to the deeper airways. Deposition efficiency (DE) is closely related to the transport mechanisms, and for nanoparticles this includes particle diameter, diffusivity and airflow rate are the governing parameters. Due to the geometric complexity of human airways, no analytical expression is available for deposition efficiency (DE).

It should be noted that, definition of the total and regional deposition fraction in human respiratory system proposed by The International Commission on Radiological Protection (ICRP) [50] also included the inhalable fraction, which was not separately defined in Eq. (8). In addition, recent studies of Koullapis et al. (2016), Naseri et al. (2014) and Li et al. (2012) noted the difference in predicting human respiratory deposition efficiency by using uniform and non-uniform inlet velocity distributions [51–53]. By enforcing mouth inlet velocity with experimental measurement, Koullapis et al. (2016) [51] predicted an enhanced deposition on the tongue with the uniform inlet velocity. However, difference in regional deposition quickly became negligible beyond the oropharynx and laryngopharynx. On the other hand, prediction by Naseri et al. (2014) [52] showed significant variation in regional particle deposition in human upper airways (up to the trachea) by using the uniform and realistic velocity profile at the nostril inlet. The difference was persistent throughout the airways.

The deposition results in the current study are the deposition fractions from inhalation exposure rates, known as aspiration efficiencies (Anthony and Flynn, 2006; Se et al. 2010) [54, 55]. Further comprehensive investigations are needed to understand the relationship between particle deposition fraction and the non-uniform flow distribution at the airway inlet.

#### Total deposition in human and rat nasal cavities

**Fig. 5:**
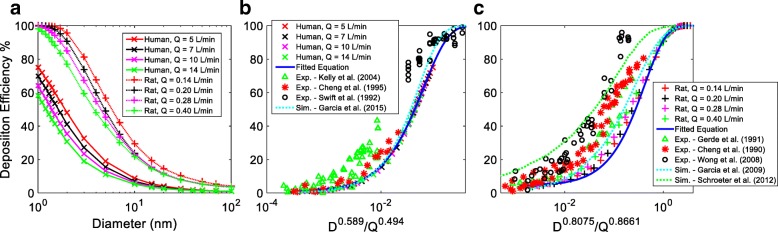
Particle deposition efficiency in human and rat nasal cavities. **a** against particle size (human and rat); **b** against *D*^*0.589*^*/Q*^*0.494*^(human); **c** against *D*^*0.8075*^*/Q*^*0.8661*^ (rat)

Figure 5 indicates the deposition efficiency of the inhaled nanoparticles (1 to 100 nm) in human and rat nasal cavities. The simulation results were plotted against particle size for breathing rates of 5, 7, 10 and 14 L/min for human, and 0.14, 0.20, 0.28 and 0.40 L/min for the rat model (Fig. 5a). The results show total deposition was inversely related to particle size and breathing rate. Larger particles and higher breathing rates resulted in lower deposition efficiency. At 1 nm diameter, particle deposition efficiency ranged from 58.3 to 75.2% for human and 100% for the rat. It decreased to about 6% (human) and 20% (rat) when particle size was increased to 10 nm. Beyond 15 nm, particle deposition efficiency was below 5% and approached 1% at 100 nm diameter for the human model, while for the rat model this was < 14% for 15 nm, and approached 3% at 100 nm. Overall, the rat model was significantly more efficient in capturing nanoparticles from the inhaled air (Fig. 5a), which was about 1.5 to 4.5 times more than in the human model.

The deposition efficiency sensitivity to flow rate had maximum variation of 13 to 19% for particles below 3 nm in the human model. For the rat model, almost all 1 nm particles were captured by the nasal cavity, while the deposition efficiency sensitivity to flow rate had maximum variation of 11 to 22% for particles in the range of 1.5 to 7 nm. In both models, the sensitivity to the flow rate decreased as particle size increased, and the flow rate became insignificant when the particle size reached 100 nm (Fig. 5a).

The data was curve fitted and correlated most efficiently with *D*^*0.589*^*/Q*^*0.494*^ for the human model, and *D*^*0.8075*^*/Q*^*0.8661*^ for the rat model (Fig. 5b and c). Here *D* is the particle diffusivity in *m*^*2*^*/s*, and *Q* is the breathing airflow rate in *m*^*3*^*/s*. The developed empirical equations for human and rat were given as:

9 $$ {DE}_{human\_ nasal\_ cavity}=\left(1-1.0013{e}^{-17.87\frac{D^{0.589}}{Q^{0.494}}}\right)\times 100 $$

10 $$ {DE}_{rat\_ nasal\_ cavity}=\left(1-0.95{e}^{-2.393\frac{D^{0.8075}}{Q^{0.8661}}}\right)\times 100 $$

In Fig. 5b and c, experimental measurements of total deposition efficiencies in the human and rat nasal cavities from literature were plotted against the derived curve fitting parameters for comparison [23–28]. These experiments were performed on nanoparticles of 0.58 to 155 nm at low to moderate breathing conditions, 4 to 12 L/min in human, and of 5 to 200 nm at breathing rates of 0.2 to 0.6 L/min in the rat. The results show that for the human model, the simulation results most closely resembled measurements from Cheng et al. (1995) [24] for larger particles at low deposition, while it matched well with the measured deposition efficiency of Swift et al. (1992) [25] for high diffusive particles. For the rat model, the simulation results most closely matched to the measurement of Gerde et al. (1991) [26]. For both human and the rat, simulation results generally followed the lower bound of the measurement data (Fig. 5b and c). The simulation agreed reasonably well with the trend in the experimental data. Variations between simulation and experimental data might be attributed to inter-subject variation between human subjects (Fig. 5b), and the differences in rat strain, mass, geometry and model resolutions (Fig. 5c). Data scattering were observed in all experiments, however within tolerance, due to noise and high sensitivity of the measurements.

Prior simulation [21, 22, 56] on human and rat nasal nanoparticle deposition was also included in Fig. 5b and c for comparison. While reasonable agreement was achieved in the human model, the current simulation results of the rat nasal cavity was slightly lower than prior results due to variation in testing subjects and simulation conditions.

#### Olfactory deposition in human and rat nasal cavities

**Fig. 6:**
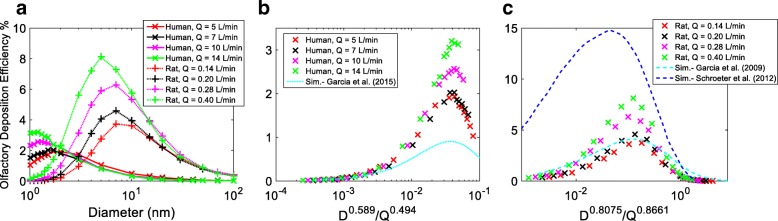
Particle deposition efficiency in human and rat olfactory mucosa. **a** against particle size (human and rat); **b** against *D*^*0.589*^*/Q*^*0.494*^ (human); **c** against *D*^*0.8075*^*/Q*^*0.8661*^ (rat)

Figure 6 shows the human and rat olfactory deposition efficiency of inhaled nanoparticles in the range from 1 to 100 nm at breathing rates of 5, 7, 10 and 14 L/min for human, and 0.14, 0.20, 0.28 and 0.40 L/min for rat, respectively. Olfactory deposition exhibited distinctive characteristics for high diffusive particles, when compared to the overall nasal deposition (Fig. 5a). Rather than reducing deposition, a higher breathing rate enhanced deposition for particles below 2.5 nm for human, and up to 70 nm for the rat respectively. This implies that, for the majority of nanoparticles considered (d < 70 nm), rat olfactory deposition favored a higher breathing rate. Furthermore, deposition increased together with the particle size until a flow dependent peak value was reached. Particle sizes corresponding to the peak deposition reduced as the breathing rate was increased. Based on the simulation, peak deposition occurred at 1.7, 1.5, 1.3 and 1.2 nm at breathing rates of 5, 7, 10 and 14 L/min for the human; whereas for the rat, this occurred at 7 nm for breathing rates of 0.14, 0.20 and 0.28 L/min, and at 5 nm for the highest breathing rate of 0.40 L/min. Overall, human and rat olfactory deposition was extremely low, < 3.5% and < 8.1%, in all cases considered. An interesting observation in Fig. 6a was that a higher percentage of high diffusive nanoparticles (d < 2 nm) reached the human olfactory mucosa compared to the rat model (2 to 80 times). Between 2 and 3 nm, the deposition percentage in both models were similar, and > 3 nm particles, there was significantly higher deposition in the rat olfactory mucosa than in the human (2 to 32 times).

Figure 6b and c presented the human and rat olfactory deposition efficiency against *D*^*0.589*^*/Q*^*0.494*^ and *D*^*0.8075*^*/Q*^*0.8661*^. Low diffusive particles corresponded well with the correlation, however, large discrepancies were observed for high diffusive particles. Clearly, more factors had an influence and olfactory deposition cannot be efficiently described in a similar manner as that in the nasal cavity. Simulation results from literature [21, 22, 56] were also plotted in Fig. 6b and c for comparison, where a similar trend was observed, although a noticeable difference was found for high diffusive particles. This was not surprising considering the high sensitivity of olfactory deposition on olfactory surface area, particularly for high diffusive particles. For the same reason, 3 models with varying olfactory sizes were considered in the work of Garcia et al. (2015) [22] for human models, and fitting was provided on Model 1A (surface area of 11.2 cm^2^ or 5.5% of the nasal cavity) which showed that the area was about half the size of the olfactory model used in this study (Fig. 1). Considering these variations, the current simulation and the prediction of Garcia et al. (2015) [22] showed excellent agreement on human olfactory deposition prediction. In the rat nasal olfactory (Fig. 6c), the current simulation agreed well with Garcia et al. (2009) [21] however, was significantly lower than the prediction from Schroeter (2012) [56]. Due to the complexity in experimental procedures, there has been no quantitative report on experimental measurement of nanoparticle deposition onto human and rat olfactory mucosa.

In summary, nanoparticle (1 to 100 nm) deposition on human and rat olfactory mucosa was extremely low, with the highest deposition (< 3.5 and 8.1%) seen for high diffusive particles around 1.5 nm and 5 nm, respectively. As particle size increased, deposition in the human and rat olfactory region decreased to 0.01 and 0.38% for 100 nm. For both species, breathing rate positively correlated with deposition of high diffusive particles; however, it negatively correlated with deposition of low diffusive particles. The deposition efficiency data for the whole nasal cavity was curve fitted and characterized by *D*^*a*^*/Q*^*b*^ with *a* and *b* being coefficients, however this was not possible when considering the olfactory region by itself. More factors need to be included and further research is needed.

Empirical correlations for olfactory deposition are proposed for human and rat nasal models, as a function of the diameter (nm) and breathing airflow rate (L/min), given as:

11 $$ {DE}_{human\_ olfactory}=\exp \left[{a}_0+{a}_1\cos \left(\omega \ln (d)\right)+{b}_1\sin \left(\omega \ln (d)\right)+{a}_2\cos \left(2\omega \ln (d)\right)+{b}_2\sin \left(2\omega \ln (d)\right)+{a}_3\cos \left(3\omega \ln (d)\right)+{b}_3\sin \left(3\omega \ln (d)\right)+{a}_4\cos \left(4\omega \ln (d)\right)+{b}_4\sin \left(4\omega \ln (d)\right)+{a}_5\cos \left(5\omega \ln (d)\right)+{b}_5\sin \left(5\omega \ln (d)\right)+{a}_6\cos \left(6\omega \ln (d)\right)+{b}_6\sin \left(6\omega \ln (d)\right)\right] $$

and

12 $$ {\displaystyle \begin{array}{l}{DE}_{rat\_ olfactory}={a}_0+{a}_1\cos \left(\omega \ln (d)\right)+{b}_1\sin \left(\omega \ln (d)\right)+{a}_2\cos \left(2\omega \ln (d)\right)+{b}_2\sin \left(2\omega \ln (d)\right)+{a}_3\cos \left(3\omega \ln (d)\right)+{b}_3\sin \left(3\omega \ln (d)\right)+{a}_4\cos \left(4\omega \ln (d)\right)+{b}_4\sin \left(4\omega \ln (d)\right)\\ {}\end{array}} $$

where, *a*_*i*_, *b*_*i*_ and *ω* were coefficients, developed as a polynomial function of the breathing airflow rate Q (L/min) in the following form:

13 $$ {c}_0+{c}_1Q+{c}_2{Q}^2+{c}_3{Q}^3 $$

**Fig. 7:**
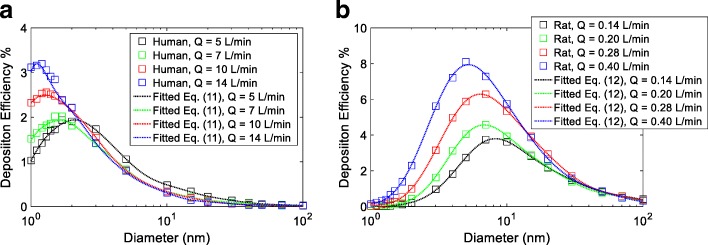
Empirical equations of particle deposition efficiency in human and rat nasal olfactory mucosa. **a** human; **b** rat

Here, *c*_0_ to *c*_3_ were empirical coefficients given in Tables 4 and 5 for human and the rat respectively. Fitting of Q in Eq. (13) correlated to 5 to 14 L/min for human and 0.14 to 0.40 L/min for the rat, and the diameter, *d* in Eqs. (11) and (12) was in nanometer (nm). Given the nanoparticle size and breathing rate, Eqs. (11) and (12) provides a practical tool to quantitatively predict nanoparticle deposition in the human and rat olfactory region (Fig. 7). For example, at a breathing rate of 7 L/min (human), the calculated *a*_*0*_*, a*_*1*_*, b*_*1*_ are − 2.0716, 0.6722, 2.6808 using Eq. (13) and Table 4. With all *a*_*i*_, *b*_*i*_ and *w* known, Eq. (11) can be used to predict human olfactory deposition efficiency for a particle of a given size. Caution should be given when predicting olfactory deposition of nanoparticles beyond the fitting conditions.

**Table 4:** Human olfactory deposition empirical coefficients for Eq. (12)

	*c*_*0*_	*c*_*1*_	*c*_*2*_	*c*_*3*_
*a*_*0*_	7.998	−3.501	0.3869	−0.01318
*a*_*1*_	5.624	−2.323	0.319	−0.0126
*b*_*1*_	−9.529	4.448	−0.5133	0.01815
*a*_*2*_	−6.719	2.673	−0.2938	0.01005
*b*_*2*_	−9.928	4.184	−0.5074	0.01866
*a*_*3*_	−9.337	3.716	−0.4336	0.01558
*b*_*3*_	1.276	−0.3492	0.02392	−0.0003864
*a*_*4*_	−2.653	1.166	−0.147	0.005602
*b*_*4*_	5.583	−2.144	0.2431	−0.008509
*a*_*5*_	2.671	−0.9673	0.1053	−0.003533
*b*_*5*_	2.934	−1.193	0.1428	−0.005245
*a*_*6*_	1.434	−0.5703	0.06723	−0.002433
*b*_*6*_	−1.004	0.3552	−0.03821	0.001264
*ω*	1.231	−0.03935	−0.001761	0.0002861

**Table 5:** Rat olfactory deposition empirical coefficients for Eq. (12)

	*c*_*0*_	*c*_*1*_	*c*_*2*_	*c*_*3*_
*a*_*0*_	2.923	−25.31	127.9	−160.3
*a*_*1*_	−2.799	20.29	− 104.4	142.1
*b*_*1*_	0.8169	−14.19	101.9	− 135.6
*a*_*2*_	0.8552	−5.735	8.181	−10.56
*b*_*2*_	−0.319	0.4386	−19.01	35.32
*a*_*3*_	−0.9219	12.02	−40.67	43.69
*b*_*3*_	−0.01631	3.591	−22.66	30.82
*a*_*4*_	0.1696	−3.974	19.8	−26.18
*b*_*4*_	−0.7907	9.622	−33.53	36.36
*ω*	0.9015	4.71	−21.71	29.74

#### Entrance profile of olfactory deposited particles

**Fig. 8:**
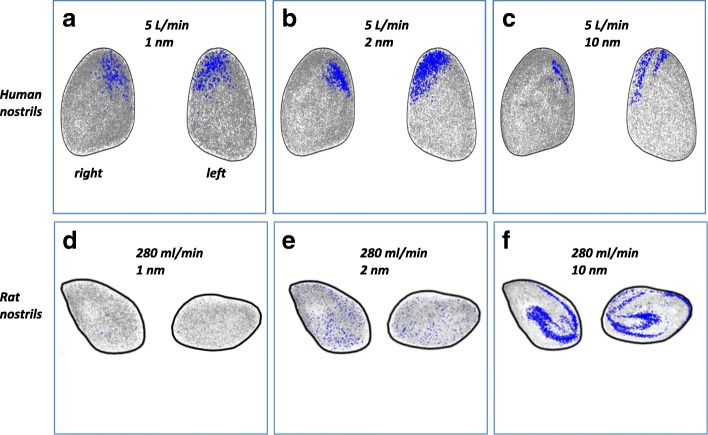
Human and rat olfactory deposited particle profiles at nostrils at breathing rate of 5 L/min (human) and 280 ml/min (rat), respectively: (**a**) 1 nm, human; (**b**) 2 nm, human; (**c**) 10 nm, human; (**d**) 1 nm rat; (**e**) 2 nm rat; (**f**) 10 nm rat

Figure 8 displays the initial locations at the nostrils of olfactory deposited particles for human and rat models at a breathing rate of 5 L/min and 280 ml/min, respectively. Inhaled nanoparticles that deposited on the nasal olfactory mucosa were plotted in blue while the rest of the particles in gray. Clear differences were observed between the models. For the human model, it was shown that olfactory deposition originated from the nose tip on the septal side, while no deposition occurred for particles inhaled via the lower half of the nostril (Fig. 8a–c). The pattern was relatively scattered for 1 and 2 nm particles, but was more concentrated in narrow bands for the 10 nm particles. For the rat model, olfactory deposited nanoparticles could originate from anywhere from the nostrils, except for the 1 nm particles where no particles reached the olfactory region (Fig. 8d–e). The figure also shows that the distribution of 2 nm deposited particles was homogeneously scattered; however for the deposited 10 nm particles, the entrance profile exhibited a swirling pattern. The information is significant to understand human and rat olfactory uptake of inhaled nanoparticles.

## Discussion

Bypassing the blood-brain barrier (BBB) by direct translocation of nanoparticles via olfactory pathway has been suspected as the cause for harmful accumulation of neurotoxicants causing progressive human cognitive impairment. With predominantly animal subjects in laboratory testing, interspecies extrapolation is important to correctly interpret cross-species data for meaningful guidance and application in human subjects. Dose-response assessment requires quantitative correlation on exposure, transport, dosimetry and genetic differences that account for interspecies variants, and equivalence metrics should be defined and evaluated. This study established empirical equations enabling quantitative assessment of nanoparticle deposition and correlation in human and rat nasal olfactory. Additionally, it allows quantification of long-term low-dose exposure to neuron toxicants that enter through the human olfactory pathway.

Significant variations in body mass and nasal\olfactory surface area between the two examined species were presented in Table 1. While body mass of the rat (Sprague Dawley of 400 g) was only 0.45% of that in human, rat olfactory surface area was about 61.71%, implying normalized olfactory sensitivity per body mass in the rat was about 137 times more advanced than that in human. On the other hand, typical breathing in resting conditions ranged from 5 to 14 L/min for human and 0.14 to 0.40 L/min for the rat [23, 46, 49], indicating the volume flow rate of inhaled air in the rat was only 2.8% of that in human.

Though possessing distinctive geometries, airflow pattern and particle flux in human and rat nasal olfactory showed comparable characteristics. In both species, the region attracted the least airflow and an extremely low number of nanoparticles passed through (Figs. 2 and 3). The phenomenon correlated to olfactory sensory physiology where as few as one odor-biding molecule was probably sufficient to activate sensing [57]. Smelling through olfactory mucosa, which immersed in extreme sedentary flow, was clearly more related to the Brownian diffusion motion. Diffusion in the region was considered the main driving force and collection of nanoparticles was size dependent. Compared to the nasal cavity, effective nanoparticle collection in the olfactory region was positively related to breathing rate, and peak deposition was around 1.2–1.7 nm for human (3.5%) and 7 nm for the rat (8.1%) (Fig. 6). Given the same exposure condition (ambient nanoparticle concentration), the rat olfactory region was significantly more efficient in collecting larger sized nanoparticles (> 3 nm); however, for extremely small sizes (< 2 nm), nanoparticle collection in the human olfactory region was considerably more efficient (Fig. 6). At comparable breathing rates of 10 L/min and 0.28 L/min for human and rat, comparisons of sample olfactory nanoparticle collection against a variety of metrics were presented in Table 6. Nanoparticle concentration of *n* particles per unit liter for each size was assumed in the calculation. It was shown from the table that total nanoparticle collection rate (#/min) was consistently higher in human than that in the rat. In the extreme case of 1 nm particles, up to 2857 times greater amount reached the human olfactory region. However, as the particle size increased, total deposition rate in the rat olfactory region quickly approached that of the human and were comparable at and beyond 20 nm. A similar trend was observed for olfactory nanoparticle deposition rate per unit surface area (#/min/mm^2^). Table 6 showed that, human and rat nasal olfactory surfaces had comparable capabilities in trapping inhaled nanoparticles except for the extremely small sizes (< 2 nm). Contrary to the prior two metrics, body mass normalized particle deposition via the nasal olfactory (#/min/kg) showed distinctive features between human and the rat. Due to significantly smaller body weight, nanoparticle deposition per unit body mass via the rat olfactory region was substantially higher than that in human. Except for the ~ 1 nm particle, the rat model showed consistently higher body mass doses (than human), from 44.8 times greater at 5 nm, quickly approaching 178.4 greater at 50 nm particle size.

**Table 6:** Comparisons of nanoparticle collection via human (10 L/min) and rat (0.28 L/min, Sprague Dawley of 400 g) nasal olfactory (ambient nanoparticle concentration: *n* particles/L for each size)

*Particle Diameter (nm)*			
	Total Collection Rate: (#/min) x n		
	Human	Rat	Rat/Human Ratio
1	0.232	0.00008	0.00035
2	0.221	0.0028	0.013
3	0.154	0.0088	0.057
5	0.081	0.0165	0.203
20	0.013	0.0082	0.633
50	0.003	0.0024	0.811
	Olfactory Surface Collection Rate: (#/min/mm^2^) x *n*		
	Human (x 1E-6)	Rat (x 1E-6)	Rat/Human Ratio
1	110.6	0.06	0.00057
2	105.4	2.15	0.02
3	73.4	6.80	0.09
5	38.6	12.74	0.33
20	6.2	6.36	1.03
50	1.4	1.88	1.31
	Collection Rate Per Body Mass: (#/min/kg) x *n*		
	Human (x 1E-4)	Rat (x 1E-4)	Rat/Human Ratio
1	26.36	2.03	0.077
2	25.11	69.4	2.77
3	17.5	220.0	12.6
5	9.20	412.0	44.8
20	1.48	205.9	139.4
50	0.34	60.8	178.4

Due to abrupt sharp turns in rat anterior nasal cavity (Fig. 2), nanoparticle uptake via the olfactory region cannot be directly correlated between the human and rat model, for the extremely small sizes (< 2 nm). The high capturing efficiency of this range of nanoparticles, at the anterior nasal channel (Figs. 4 and 5), resulted in overall significantly higher deposition rate in human, against all examined metrics, than that in the rat (Table 6). The variation was induced by anatomic difference between the two species. For larger sizes, the correlation between human and the rat in olfactory nanoparticle deposition followed a more predictable trend, where total and surface deposition rate were comparable, and body mass doses were substantially higher in the rat.

Finally, the inlet profiles of the olfactory deposited nanoparticles were quite distinct between human and the rat subjects (Fig. 8). For the human model, all olfactory deposited nanoparticles originated through the nose tip in the upper and septal corners. However, for the rat model, there was no preferential location of the olfactory deposited particles at the nostrils. The swirling patterns for the larger sized nanoparticles (10 nm) at the nostrils were more likely to be related to the downstream mixing and rotation caused by the sharp turns of the rat airway at the anterior nasal cavity (Fig. 2). This information is of significant value to understand the different characteristics of human and rat olfactory uptake of inhaled nanoparticles.

### Limitations and future study

While the current study proposed a complete computational analysis leading to the development of the empirical equations for nanoparticle deposition prediction in the human and rat nasal olfactory, there are a few limitations.

- The current study focused on only one single nasal passage sample for both species (48-year old male Asian and 12-week old male Sprague-Dawley rat), where morphological differences between individual models were not accounted. Inter- and intra-subject variability could lead to differences in regional dose exposure within the nasal passages.
- The human olfactory particle deposition around 1–2 nm size range could be extremely sensitive to the olfactory mucosa surface area (Fig. 4). A slight increase or decrease in size of the olfactory mucosa could induce significant variation in the predicted deposition rate. More data on precise location of the human olfactory epithelium in the nasal cavity is needed.
- The current study employed a steady inhalation breathing model with the assumption that particle deposition mainly occurs during the inhalation phase. The effect of the cycling breathing pattern towards olfactory nanoparticle deposition should be further evaluated.
- Only spherical solid particles were considered in the current study, while particles of other morphologies were not included.
- Fitting conditions for the current developed empirical equations were 5 to 14 L/min for human and 0.14 to 0.40 L/min with nanoparticles ranged from 1 to 100 nm. Caution should be given when predicting olfactory deposition of nanoparticles beyond the fitting conditions.
- Very few studies can be found in literature which focused on nanoparticle deposition in nasal olfactory region. In particular, there has been no reported experimental measurement of quantitative data in this respect, which is greatly needed.

## Conclusions

Regional deposition dosage of inhaled nanoparticles in the human and rat olfactory region was governed by particle size and the breathing rate (Eqs. 11–13), and interspecies correlation should be comprehensively determined by combined effect of deposition dose, physical and geometric features. The developed empirical equations (Eqs. 11–13) provided a tool to quantitatively predict inhaled nanoparticle dose onto human and the rat nasal olfactory, and laid the ground work for correlation between the two species. It also constitutes the technical basis in the fulfillment of risk assessment of airborne UFPs. Furthermore, analysis of the accumulation of long-term and low-dose exposure to exogenous nanoparticles through olfactory pathway, susceptible for progressive Parkinson’s and Alzheimer’s diseases, was made possible and will be evaluated in future studies.

## Abbreviations

BBB: Blood-brain barrier
CFD: Computational fluid dynamics
CSF: Cerebrospinal fluid
CT: Computed tomography
DE: Deposition efficiency
DPM: Discrete phase model
MRI: Magnetic resonance imaging
SPECT: Single photon emission computed tomography
